# Differential Effects of Very-Low-Volume Exercise Modalities on Telomere Length, Inflammation, and Cardiometabolic Health in Obese Metabolic Syndrome Patients: A Subanalysis from Two Randomized Controlled Trials

**DOI:** 10.3390/antiox12101847

**Published:** 2023-10-11

**Authors:** Dejan Reljic, Adriana Koller, Hans J. Herrmann, Arif B. Ekici, Markus F. Neurath, Yurdagül Zopf

**Affiliations:** 1 Department of Medicine 1, University Hospital Erlangen, Friedrich-Alexander University Erlangen-Nürnberg, 91054 Erlangen, Germany; hans.herrmann@uk-erlangen.de (H.J.H.); markus.neurath@uk-erlangen.de (M.F.N.); yurdaguel.zopf@uk-erlangen.de (Y.Z.); 2 Hector-Center for Nutrition, Exercise and Sports, Department of Medicine 1, University Hospital Erlangen, Friedrich-Alexander University Erlangen-Nürnberg, 91054 Erlangen, Germany; 3 German Center Immunotherapy (DZI), University Hospital Erlangen, Friedrich-Alexander University Erlangen-Nürnberg, 91054 Erlangen, Germany; 4 Institute of Genetic Epidemiology, Medical University of Innsbruck, 6020 Innsbruck, Austria; adriana.koller@i-med.ac.at; 5 Institute of Human Genetics, University Hospital Erlangen, Friedrich-Alexander University Erlangen-Nürnberg, 91054 Erlangen, Germany; arif.ekici@uk-erlangen.de

**Keywords:** telomeres, cellular age, high-intensity interval training, resistance training, electromyostimulation, metabolic diseases, oxidative stress, inflammatory markers, anti-aging

## Abstract

Oxidative stress (OS) and inflammation are features of metabolic syndrome (MetS) that can contribute to the shortening of telomere length (TL), a marker of cellular ageing. Research indicates that exercise can positively influence MetS-associated conditions and TL. However, the effects of low-volume exercise types on TL are still unknown. We investigated the impact of very-low-volume high-intensity interval training (LV-HIIT), one-set resistance training (1-RT), and whole-body electromyostimulation (WB-EMS) on TL, inflammation, and cardiometabolic indices in 167 MetS patients. Data were derived from two randomized controlled trials where patients were allocated to an exercise group (2 sessions/week, for 12 weeks) or a control group. All groups received standard-care nutritional weight loss counselling. TL was determined as the T/S ratio (telomere to single-copy gene amount). All groups significantly reduced body weight (*p* < 0.05), but the T/S-ratio (*p* < 0.001) only increased with LV-HIIT. OS-related inflammatory markers (C-reactive protein, interleukin-6, and lipopolysaccharide-binding protein) only decreased (*p* < 0.05) following LV-HIIT. The MetS severity z-score improved with LV-HIIT (*p* < 0.001) and 1-RT (*p* = 0.014) but not with WB-EMS. In conclusion, very-low-volume exercise modalities have differential effects on telomeres, inflammation, and cardiometabolic health. Only LV-HIIT but not strength-based low-volume exercise increased TL in MetS patients, presumably due to superior effects on OS-related inflammatory markers.

## 1. Introduction

Metabolic syndrome (MetS) is an obesity-related cluster of cardiometabolic issues, including excess abdominal fat storage, hypertension, hyperglycaemia, dyslipidemia, and insulin resistance [1]. Globally, the prevalence rates of MetS have been increasing significantly over the past few decades [2], with the latest estimates reporting that approximately 13% to 31% of the adult population is currently affected worldwide [3]. Recent data indicate that social-distancing measures related to the COVID-19 pandemic, such as quarantines, lockdowns, and closures of sports facilities, have further contributed to the spread of obesity and MetS around the globe [4,5,6]. This trend is worrying because the presence of MetS has multiple detrimental health consequences, including a substantially increased risk of cardiovascular disease [7], diabetes mellitus type 2 [8], and different types of cancers [9]. There are several factors that contribute to the pathophysiology of MetS, but increased oxidative stress (OS) and inflammation have been shown to play a crucial role in the development of cardiometabolic disorders [10]. In adipose tissue, there is an excessive release of proinflammatory mediators such as interleukin-1β (IL-1 β), interleukin-6 (IL-6), or C-reactive protein (CRP), which in turn enhance the production of reactive oxygen species [11]. Inflammation and OS have not only been identified as key precursors of MetS-associated complications but also are also considered to promote cellular ageing [12,13]. Accordingly, it has been observed that individuals with MetS exhibit a significantly shorter telomere length (TL) compared to healthy populations [14,15].

Telomeres are certain DNA-protein structures located at the end of the chromosomes and play a key role in the protection and stabilization of genomes. Telomeres typically shorten with age at each cell division, and when they reach a critical length, cells become senescent or dysfunctional. Thus, TL is considered a major biomarker of biological age [16]. Apart from the normal ageing process, there are several other factors that have been identified to promote telomere shortening, such as smoking and alcohol abuse, environmental pollution, psycho-emotional stress, and diseases associated with chronic low-grade inflammation and OS [13,15,16]. On the contrary, healthy dietary patterns [17] and regular physical activity [18] have shown their potential to preserve or even increase TL. A recent meta-analysis has reported, for example, that master athletes exhibit longer TL compared to age-matched non-athletes [19]. Werner et al. [20] have demonstrated in a randomized controlled trial (RCT) that 6 months of cardiovascular exercise, consisting of 3 weekly 45 min sessions of either aerobic endurance or interval training, increased TL in a cohort of previously inactive but otherwise healthy individuals.

Nevertheless, in spite of the fact that the manifold health benefits of regular physical activity and exercise are extensively documented through decades of research [21] and well-known by the public, a large proportion of adults [22], particularly those with obesity [23], do not meet the physical activity recommendations of a minimum of 2.5 h of moderate or, alternatively, 75 min of more intense aerobic activity within a week [24]. Large-scale investigations have constantly shown that “lack of time” is among the most commonly cited barriers to exercise, regardless of age, gender, socio-economic background, and health status [25,26,27]. Consequently, in recent years, there has been growing interest in designing more time-saving exercise programs that can be more easily incorporated into daily routines [28].

Among these, very-low-volume high-intensity interval training (LV-HIIT) [29,30], one-set resistance training (1-RT) [31], and whole-body electromyostimulation (WB-EMS) [32] have received particular attention during the last years due to their exceptional time-efficiency. Research from our laboratory and other groups has demonstrated that low-volume exercise approaches can provide several important health benefits to clinical populations, including improved cardiorespiratory fitness (CRF) [33,34,35,36,37,38,39], blood pressure (BP) [33,34,35,36,37,40,41], glycaemic control [34,37,38,39,40,42,43], liver health [37,38], inflammation status [33], body composition [31,32,33,34,35,36,37,39,40,41,43,44,45], and self-reported quality of life [35,36,41]. Notably, it was observed that very-low-volume exercise modalities differed in their beneficial effects on various physiological outcomes, with LV-HIIT, for example, showing superior efficacy in improving cardiometabolic risk indices [33,41] and inflammation outcomes [33] when compared to low-volume muscle-strengthening exercise protocols.

To date, however, it is still unknown whether very-low-volume exercise modalities may have the potential to induce an impact on TL in clinical populations. Given the increasing popularity and application of low-volume exercise routines worldwide, including clinical settings, the present study aimed to investigate and compare the effects of three popular exercise types, namely LV-HIIT, 1-RT, and WB-EMS, on TL in a cohort of obese MetS patients by analysing data from two previous RCTs. Based on data from a previous study comparing the effects of higher-volume exercise protocols on TL in a healthy population [20] and the results of our own research, indicating differential effects of very-low-volume exercise types on specific physiological parameters such as inflammatory markers related to OS [33], we hypothesized that LV-HIIT would provide superior effects on TL compared to very-low-volume muscle strengthening exercises (i.e., 1-RT and WB-EMS) in obese MetS patients.

## 2. Materials and Methods

### 2.1. Study Design and Patients

This study was a subanalysis of two RCTs in which patients were randomly allocated to different exercise interventions, including LV-HIIT (trial 1, ClinicalTrials.gov: NCT03306069), 1-RT, and WB-EMS (trial 2, ClinicalTrials.gov: NCT03306056), each lasting 12 weeks, or to non-exercising control groups (CON, trial 1 and trial 2). Both RCTs included a comparable cohort of MetS patients and followed similar study designs, with the exception of the respective exercise protocols. The LV-HIIT group in the present study was merged from two different LV-HIIT groups in trial 1 (i.e., heart rate- and lactate-threshold-based interval training prescriptions). Given that both produced similar physiological demands and improvements in CRF and cardiometabolic health [34], the data of the two groups were pooled together. Likewise, the CON group data were merged from the control patients of trial 1 and trial 2. All groups received nutritional counselling as standard of care to support weight loss through caloric restriction.

Details of the study design, sample size calculation, and randomization procedures have been previously reported for each trial [34,36,41]. Randomization in both trials was performed using a computerized random number generator (MinimPy version 3.0) [46], independently of the researchers who collected data. Prior to randomization, patients were stratified based on their maximal oxygen uptake (VO_2max_), sex, age, and body mass index (BMI) to achieve homogenous groups. The primary outcome of the present subanalysis was TL. Secondary outcomes were specific inflammatory markers and cardiometabolic outcomes, the metabolic syndrome severity score (MetS z-score), and anthropometric variables. All patients were fully informed about the scope and the experimental procedures of this investigation, which conformed to the Declaration of Helsinki, and signed an informed consent form prior to enrollment. The study protocols of the main trials were authorized by the Medical Ethical Committee of the Friedrich-Alexander University Erlangen-Nürnberg (approval numbers: 203_17B and 210_17B, respectively).

Recruitment procedures were previously described in detail [34,36,41]. Briefly, all patients were initially screened to determine whether they met eligibility criteria, which were as follows: being obese (BMI ≥ 30 kg/m^2^) and over the age of 18 years, having a clinical diagnosis of MetS based on the diagnostic criteria established by the National Cholesterol Education Program Adult Treatment Panel III (NCEP ATP III) [47], and leading a predominantly sedentary lifestyle, as previously defined [48]. Furthermore, to be included in the present study, patients had to agree to receive an additional blood draw for the determination of TL. Patients were not eligible for study participation if they met any of the following exclusion criteria: pregnancy, clinical diagnosis of heart disease, cancer, or any significant internal or musculoskeletal condition that may preclude safe engagement in a training program. The included patients consented to maintain their regular lifestyle throughout the study in order to avoid any confounding factors. Only patients who completed at least 80% of the scheduled exercise sessions were considered for the final analysis.

### 2.2. Pre- and Post-Intervention Examinations

A week before the onset of the intervention period, patients received the first examination (T-1). The examination included measurements of all study outcomes, as specified in more detail below. Additionally, electrocardiography in rest and during exercise, as well as evaluation of routine blood and urine parameters, were performed to rule out clinical contraindications for participating in an exercise program. The follow-up examination was conducted during the first post-intervention week (T-2) at a similar daytime to reduce potential circadian variability. At both examinations, patients were asked to show up in an overnight-fasted state and to abstain from alcohol and vigorous activities for a minimum of 24 h before their visit. The measurements took place under stable ambient conditions (temperature: 22–24 °C; humidity: 30–50%) and in a strictly standardized setting at our research laboratory. All investigators involved in data collection were blinded to patients’ group assignment.

#### 2.2.1. Measurement of Blood Pressure

Upon arriving at the laboratory and prior to the measurements, patients were asked to empty their bladder. Subsequently, BP was measured after a 5 min resting period in a sitting position using an automatic upper arm BP monitor (M5 professional, Omron, Mannheim, Germany). In accordance with recent guidelines [49], the measurements of systolic (SBP) and diastolic (DBP) BP were carried out two consecutive times on both arms, with a break of 60 s between each measurement. The mean values of the arm with the higher BP values were used for further analysis and for the calculation of mean arterial blood pressure (MAB) according to the following equation:

(1/3 [SBP − DBP]) + DBP

#### 2.2.2. Sampling of Blood and Analyses

Following BP assessment, patients stayed seated and venous blood was collected from puncture of an antecubital vein into vacutainer tubes (Sarstedt, Nümbrecht, Germany). One part of the blood samples was immediately transferred to the Central Laboratory of the University Hospital Erlangen for the analysis of CRP, glucose (GLU), triglycerides (TG), total cholesterol (TC), and low-density (LDL) and high-density lipoprotein cholesterol (HDL) using a photometrical or turbidimetric (CRP) determination method (Clinical Chemistry Analyzer AU700 or AU5800, Beckman Coulter, Brea, CA, USA; coefficients of variation (CV): 1.1–1.4%), and glycated haemoglobin A_1c_ (HbA_1c_) using turbidimetric immunoassays (COBAS Integra 400, Roche Diagnostics, Mannheim, Germany; CV: 2.7%).

Interleukine-1 beta (IL-1β), interleukine-6 (IL-6), interfereonegamma (IFNγ), adiponectin, and lipopolysaccharide-binding protein (LBP) were measured at a laboratory in our research centre. All analyses were conducted with enzyme-linked immunosorbent assays (Human IFNγ DuoSet ELISA; Human IL-1β/IL-1F2 DuoSet ELISA; Human IL-6 Quantikine HS ELISA Kit; Human LBP DuoSet ELISA; all R&D systems, Wiesbaden, Germany) according to instruction of manufacturers. In each analysis, 100 µL non-diluted serum was applied, and analyses were executed twice. In brief, well-plates were covered with the respective capture antibody at room temperature overnight. Three washing steps were performed in total, followed by reagent dilution blocking for 1 h at room temperature. Subsequently, 100 µL of undiluted serum samples was added in duplicates to the coated well-plates for 2 h at room temperature. Thereafter, well-plates were rinsed and incubated with a biotinylated detection antibody for another 2 h at room temperature. Bound antibodies were quantified using a Streptavidin-Horseradish Peroxidase (HRP) (Thermo Fisher Scientific Inc., Waltham, MA, USA) solution for 20 min at room temperature, and colour development was conducted with a substrate solution for an additional 20 min. The procedure was stopped with sulphuric acid, and the optical density was determined at 450 nm using iMark^TM^ Microplate Reader (Bio-Rad, Hercules, CA, USA). Finally, serum concentrations were obtained using point-to-point computation.

Another collection tube containing ethylenediaminetetraacetic acid (EDTA) was used for the determination of TL and stored at −80° until later analysis at the Institute of Human Genetics of the University Hospital Erlangen.

#### 2.2.3. Determination of Telomere Length

Relative telomere length was assessed using quantitative polymerase chain reaction (qPCR) in a multiplex approach, enabling the amplification and detection of both the telomere product and the single-copy gene product (albumin) in a single run. The exact method and details of the calculation have been previously described [50]. Briefly, all samples were measured in triplicates, and each 10 µL reaction contained 1 µL DNA (20 ng), 5 µL PowerUp™ SYBR™ Green Master Mix (Thermo Fisher Scientific, Waltham, MA, USA), 700 nM telomere primers, and 400 nM albumin primers. The telomere and albumin primer sequences were telg (5′-ACACTAAGGTTTGGGTTTGGGTTTGGGTTTGGGTTAGTGT), telc (5′-TGTTAGGTATCCCTATCCCTATCCCTATCCCTATCCCTAACA), albugcr1 (5′-CGGCGGCGGGCGGCGCGGGCTGGGCGGAAACGCTGCGCAGAATCCTTG), and alb-dgcr1 (5′-GCCCGGCCCGCCGCGCCCGTCCCGCCGCTGAAAAGTACGGTCGCCTG). All qPCR measurements were performed on a Quantstudio 6 flex system (software version 1.7.1, Thermo Fisher Scientific, Waltham, MA, USA). The raw data from each well underwent efficiency correction using the LinRegPCR software (version 2017.1) [51] to calculate individual PCR efficiency for both targets. A CV of <5% was set as requirement to keep the sample. For this study, the quality criteria for measurement of relative TL were met in all samples with available DNA. Subsequently, the following equation was used to calculate the fraction efficiency (eff) of the samples to the threshold cycle (Ct) value of the calibrator:

$$Relative\;T/S\;Ratio=\frac{eff{\left(tel,sample\right)}^{Ct(tel,sample)}}{eff{\left(alb,sample\right)}^{Ct(alb,sample)}}/\frac{eff{\left(tel,calibrator\right)}^{Ct(tel,calibrator)}}{eff{\left(alb,calibrator\right)}^{Ct(alb,calibrator)}}$$

With this approach, the final relative TL was determined via the ratio of the telomere product versus a single-copy gene (T/S ratio). For calculation of the T/S ratio, an intern control was used as a calibrator, defined as T/S ratio = 1. In total, four different positive controls were included as additional references: a manufactured DNA (commercially available DNA; Human Genomic DNA, Roche, Merck KGaA, Darmstadt, Germany) and three control samples that were used for comparison of the inter-assay variability. The four positive controls were included on all plates, and the respective T/S ratios were compared for the entire study after finishing all measurements (CV: 2.26–5.0). All analyses for TL determination were performed blinded to the patients’ group assignment.

#### 2.2.4. Measurement of Anthropometric Values and Body Composition

Waist circumference (WC) was assessed to the closest cm, in the standing position in the middle area of the iliac crest and the last palatable rib, using a flexible measuring tape (Seca, Hamburg, Germany). Stature was measured to the closest cm with the help of a standard stadiometer (seca 274, Seca GmbH & Co., KG, Hamburg, Germany). Patients’ weight was measured to the closest 0.1 kg using the seca mBCA 515 device (Seca, Hamburg, Germany). Body composition was evaluated using segmental multi-frequency bioelectrical impedance analysis (BIA) (seca mBCA 515, Seca, Hamburg, Germany). The validity of the BIA unit for body composition determination has been previously reported [52].

#### 2.2.5. Determination of the Metabolic Syndrome Severity Score

The MetS z-score was computed individually for males (M) and females (F) according to the formulas by Johnson et al. [53] as follows:

M: [(MAB − 100)/9.1] + [(WC − 102)/7.7] + [(GLU − 100)/11.3] + [(40 − HDL)/9.0] + [(TG − 150)/81.0]

F: [(MAB − 100)/9.1] + [(WC − 88)/9.0] + [(GLU − 100)/11.3] + [(50 − HDL)/14.1] + [(TG − 150)/81.0]

The MetS z-score is a continuous risk score used to estimate MetS severity and is regarded as more accurate for predicting cardiometabolic risk of a patient in comparison to separate analyses of individual MetS components [54].

#### 2.2.6. Cardiopulmonary Exercise Testing

A standardized ramp exercise protocol was carried out on a stationary cycle ergometer (Corival cpet, Lode, Groningen, The Netherlands) to measure patients’ VO_2max_, ventilatory threshold (VT), maximum power output (W_max_), and maximum heart rate (HR_max_). After a brief familiarization phase, the protocol started with a workload of 50 W and then gradually increased at rate of 12.5 W/min in women and 15 W/min in men, respectively, until volitional exhaustion. The cardiopulmonary exercise test (CPET) was typically completed within 8–12 min until patients reached exhaustion. Heart rate was continuously recorded during the exercise test via a 12-lead electrocardiography monitor (custo cardio 110, custo med, Ottobrunn, Germany). Ventilatory variables, including ventilation, oxygen uptake, and output of carbon dioxide, were measured with an automated metabolic cart (Metalyzer 3B-R3, Cortex Biophysik, Leipzig, Germany), with all recordings averaged at intervals of 10 s. Attainment of VO_2max_ was confirmed by the occurrence of a minimum of two of the listed criteria [55]: plateau in oxygen consumption, 90% of the age-predicted HR_max_ (according to the equation: 220–age), peak respiratory exchange ratio (RER) ≥ 1.10, and rating of perceived exertion of ≥19 on the Borg scale [56]. Submaximal exercise capacity was assessed by the power output at the VT (W_VT_), which was detected using the V-slope method introduced by Beaver et al. [57].

### 2.3. Analysis of Food Intake and Nutritional Counseling

Patients were instructed to record their daily nutritional intakes within the respective weeks prior to T-1 and T-2 for three days in a row with the help of a standardized food protocol (Nutri-Science, Freiburg, Germany), which is well established and widely used in clinical routine [58]. The completed food records were analysed using the software PRODI 6 expert version (Nutri-Science, Freiburg, Germany) in order to quantify patients’ average calorie and macronutrient intakes. Based on the food record data, patients received individual one-on-one nutritional counselling from a certified dietitian. International guidelines for the treatment of obesity aim for patients to achieve a daily caloric restriction of −500 kcal from their habitual energy intake [59]. In addition, patients received instruction to maintain a protein intake of ≥1.0 g/kg/day to counteract muscle mass reduction during caloric restriction [60]. To assist the practical implementation of the calorie-reduced diet and to support adherence, respectively, patients were handed out information sheets with specific recipes and food lists.

### 2.4. Exercise Protocols

All training sessions were conducted in the Training Center of the Hector-Center for Nutrition, Exercise and Sports and were supervised by certified physiotherapists and sports therapists in a 1:1 (WB-EMS) or 1:2 (LV-HIIT and 1-RT) therapist–patient ratio. For patients’ convenience, the training sessions could be scheduled individually at preferred timeslots during the opening hours of the Training Center. Each exercise protocol was completed two times per week, with a minimum of 2 recovery days between the training sessions, for a period of 12 weeks.

#### 2.4.1. Very-Low-Volume High-Intensity Interval Training

The LV-HIIT protocol was performed on cycle ergometers (Corival cpet, Lode, Groningen, The Netherlands), as introduced by Reljic et al. [61]. Each session involved a brief 2 min warm-up phase, followed by five exercise intervals of 1 min duration at 80–95% HR_max_ interspersed by 1 min of low-intensity recovery, and a concluding 3 min cool-down period, resulting in a total time commitment of 14 min/session. The individual target heart rate ranges were determined based on the cardiopulmonary exercise test results. Patients were provided with a chest belt heart rate monitor (acentas, Hörgertshausen, Germany), which allowed them to follow their heart rate on a screen throughout the exercise session. During the interval bouts, patients were directed to accelerate the cadence and/or to increase the resistance of the ergometer to achieve their target heart rate. During warm-up, the active recovery periods, and cool-down, patients cycled at a self-selected low-intensity resistance and pace. The initial minimum exercise intensity to be achieved during each interval was chosen to correspond to the recommended lower heart rate threshold for HIIT (i.e., 80% of HR_max_) [62]. To provide a progressive training stimulus, the target heart rate zones for the intervals were adjusted upwards during the intervention period as follows: week 1–4: 80–85% HR_max_, week 5–8: 85–90% HR_max_, and week 9–12: 90–95% HR_max_.

#### 2.4.2. One-Set Resistance Training

All 1-RT sessions started with a short warm-up of low-intensity ergometer cycling for 5 min. The main part of the session involved five resistance exercises to address the major muscle groups (abdominals, lower back, legs, chest, and upper back) with the following exercise machines: abdominal crunch, lower back machine, leg press, chest press, and latissimus pulldown machine (TechnoGym, Neu-Isenburg, Germany). For each exercise, a single set of exercise was performed until volitional failure. Each repetition was performed with a 2 s concentric and 2 s eccentric phase of muscle work. The weight load for each exercise was determined based on a maximum strength testing (one-repetition maximum, 1RM) that was performed at T-1 and every 4 weeks of the intervention period, as previously described in detail [41]. As recommended for previously untrained individuals, a moderate weight load of 50–60% of 1RM was initially used for each exercise to accustom patients to RT [63]. According to RT guidelines and recent recommendations [63,64,65], the weight load was progressively increased in 4-week cycles using the following pattern: 50–60% 1RM during weeks 1–4; 60–75% 1RM during weeks 5–8; and 70–80% 1RM during weeks 9–12. The average total time effort per session was ~15 min (excluding warm-up).

#### 2.4.3. Whole-Body Electromyostimulation Exercise

Similar to the 1-RT protocol, patients first warmed up for 5 min on a stationary cycle ergometer prior to each WB-EMS session. Subsequently, patients were equipped with a specific vest, upper arm and thigh cuffs, and a hip belt with built-in electrodes that were connected to the WB-EMS device (Miha Bodytec, Gersthofen, Germany). As previously described and typically used in fitness clinical settings [66,67], the electrical muscle stimulation was implemented according to the following protocol: bipolar electric current, 85 Hz impulse frequency, 350 s pulse width, 6 s of stimulation, interspersed by 4 s of rest, for a total duration of 20 min. During each stimulation phase, eight muscle groups (i.e., upper arms, chest, upper back, latissimus, abdomen, lower back, buttocks, and thighs) were simultaneously addressed. To support the activation of the target muscle groups, the electrical stimulation was combined with light dynamic movements, including moderate squats, butterfly movements, latissimus pull-down movements, and trunk flexions/extensions, each performed twice with 10 repetitions. During each session, the current intensity was individually adapted to produce a perceptible muscle contraction and carefully increased based on the patient’s feedback. In contrast to conventional RT, there are no objective intensity ranges for WB-EMS; the training intensity progression was based on rates of perceived exertion. As proposed by recent guidelines [68], patients started with a moderate impulse intensity (i.e., “4” = somewhat strong on the modified Borg CR10 Scale [69]) during the first 4 weeks, which was increased to “5” (=hard) during weeks 5–8 and “6” (=hard+) during weeks 9–12 in order to provide training load progression.

### 2.5. Statistical Analysis

Statistical analysis was performed using SPSS version 24.0 (IBM Corp., Armonk, NY, USA). All descriptive data and changes between T-1 and T-2 are reported as means with 95% confidence intervals (95% CI). The Kolmogorov–Smirnov test with Lilliefors correction was used to test the normality of the data, and variance homogeneity was confirmed using Levene’s test. Main effects of group, time, and interaction were evaluated using a 4 × 2 repeated-measures analysis of variance (ANOVA) or analysis of covariance (ANCOVA). Additionally, ANCOVAs were performed to check whether age, sex, or educational level had any influence on changes in TL, cardiometabolic, and inflammation outcomes. In case significant group, time, or interaction effects were detected, 1-way ANOVAs with subsequent Holm–Sidak’s post hoc tests were performed to analyse for between-group differences, and post hoc paired *t*-tests were conducted to determine within-group (pre-post) changes, respectively. In the case of skewed data, log or square root transformation was performed, and subsequently, the same statistical tests were applied to the transformed values. If data transformation did not address the violations to meet assumptions of parametric tests (GLU, HDL, HbA_1c_), a Friedman 2-way analysis of variance by ranks was used on the non-transformed data, followed by Kruskal–Wallis tests and Dunn’s Bonferroni post hoc tests for comparisons between groups, and Wilcoxon’s tests for comparisons within groups, respectively. Effect sizes were determined using eta-squared (*ɳ*^2^, small effect: ≤0.01, medium effect: ≥0.06, and large effect: ≥0.14) for all ANOVAs, and Kendall’s coefficient of concordance (*W*, small effect: ≥0.10, medium effect: ≥0.30, and large effect: ≥0.50) for the Friedman tests [70]. The level of significance was defined as *p* < 0.05 for all statistical tests performed.

## 3. Results

### 3.1. Study Flow, Training Data, Dietary Intakes, and Adverse Events

In total, 194 obese MetS patients (trial 1: *n* = 105, trial 2: *n* = 89) were randomized into the different low-volume exercise and control groups. From this pool of patients, 167 agreed to the additional blood draw for the determination of TL. During the intervention period, 39 patients dropped out, and 5 patients were excluded from the analysis due to insufficient compliance. Specific reasons for dropout in both trials were previously reported [34,36,41]. Thus, the sample for this subanalysis involved a total of 123 patients. Patients’ baseline characteristics are shown in Table 1.

No significant differences were seen among the four groups for the primary and secondary outcomes at T-1 (Table 1), and there were no significant differences regarding the average age (*p* = 0.745), sex distribution (*p* = 0.377), and educational level (*p* = 0.850). No significant effects of age, sex, and educational level on changes in TL, cardiometabolic, and inflammation markers were detected (all *p*-values > 0.05), except for a significant influence of sex on the decrease in waist circumference (*p* = 0.016). Therefore, the data of female and male patients were analysed together. The attendance rates were high and similar in all exercise groups (LV-HIIT: 94 ± 7%, 1-RT: 94 ± 6% and WB-EMS 94 ± 6%). The recorded training data confirmed that the targeted exercise intensity was reached in all exercise groups. In the LV-HIIT group, the average heart rate values during the interval bouts and recovery periods were 91 ± 6% of HR_max_ and 69 ± 6% of HR_max_, respectively. In the 1-RT and WB-EMS group, the average exercise intensity corresponded to 66 ± 3% of 1RM and 5.6 ± 1.3 on the Borg CR10 Scale, respectively. There were no significant differences in dietary intakes among the four groups at T-1 (Table 1). In accordance with the standard-care nutritional counselling, all groups significantly reduced daily energy intake from T-1 to T-2 (LV-HIIT: −305 kcal, 95% CI −609 to –2 kcal, *p* = 0.024; 1-RT: –511 kcal, 95% CI −936 to −86 kcal, *p* = 0.011; WB-EMS: −514 kcal, 95% CI −833 to –196 kcal, *p* = 0.002; CON: −418 kcal, 95% CI −819 to −17 kcal, *p* = 0.021), without significant group differences. In both trials, no adverse events occurred during the study period that were related to the exercise protocols.

### 3.2. Telomere Length

**Figure 1:**
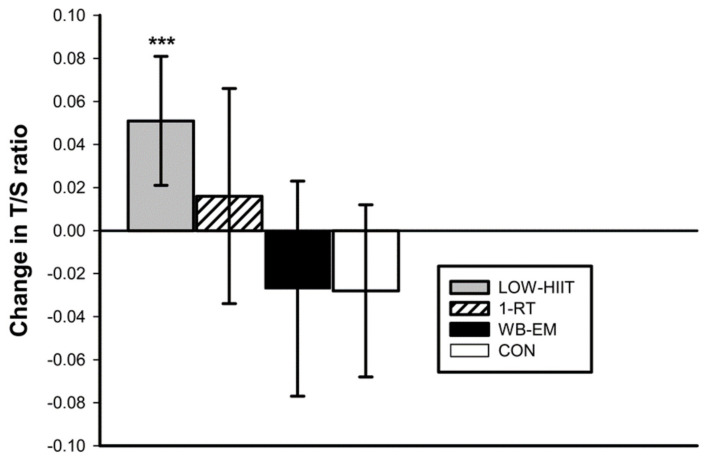
Changes in T/S ratio following the 12-week intervention. *** (*p* < 0.001) denotes significant change between pre- and post-intervention.

The baseline T/S ratios were not significantly different (*p* = 0.097) between the four groups, with mean values of 0.79 ± 0.15 for the LV-HIIT group, 0.82 ± 0.13 for the 1-RT group, 0.85 ± 0.14 for the WB-EMS group, and 0.86 ± 0.15 for the CON group. After the 12-week intervention period, a significant group-by-time interaction was identified (*p* < 0.015, η^2^ = 0.08). Post hoc tests revealed a significant increase in the T/S ratio in the LV-HIIT group by 0.05 (95% CI: 0.02 to 0.08, *p* < 0.001), while no significant changes were observed in the other groups (Figure 1).

### 3.3. Inflammatory Markers

Significant group-by-time interactions were found for CRP (*p* = 0.048, η^2^ = 0.08) and IL-6 (*p* = 0.039, η^2^ = 0.09), and a significant main effect of time was detected for LBP (*p* = 0.043, η^2^ = 0.05). The post hoc test showed that CRP, IL-6, and LBP dropped significantly in the LV-HIIT group. Compared to the CON group, reductions in CRP and IL-6 were significantly larger in the LV-HIIT group. In all other groups, inflammatory markers did not change significantly between T-1 and T-2 (Table 2).

### 3.4. Anthropometric Variables

Significant main effects of time were observed for body weight (*p* < 0.001, η^2^ = 0.36), BMI (*p* < 0.001, η^2^ = 0.37), percentage of body fat (*p* < 0.001, η^2^ = 0.20), and WC (*p* < 0.001, η^2^ = 0.33). Further, a significant group-by-time interaction was found for WC (*p* = 0.006, η^2^ = 0.10). In addition, a significant sex difference in the change in WC was detected (*p* = 0.016), with men showing a greater decrease than women (−2.6, 95% CI −4.6 to −0.6 cm, *p* = 0.010). As shown in Table 3, all groups significantly reduced body weight and body fat. There were no significant differences in the magnitude of body weight loss between groups. Waist circumference decreased significantly in the exercise groups but not in the CON group. The WC reduction was significantly greater in the LV-HIIT group in comparison to the CON group (*p* = 0.005).

### 3.5. Cardiometabolic Variables

Significant main effects of time and group-by-time interactions were detected for SBP (*p* < 0.001, η^2^ = 0.11 and *p* = 0.002, η^2^ = 0.12, respectively), DBP (*p* = 0.022, η^2^ = 0.04 and *p* < 0.001, η^2^ = 0.15, respectively), MAB (*p* < 0.001, η^2^ = 0.11 and *p* < 0.001, η^2^ = 0.17, respectively), and the MetS z-score (*p* < 0.001, η^2^ = 0.22 and *p* < 0.001, η^2^ = 0.13, respectively). Further, a significant main effect of time was found for HbA_1c_ (*p* = 0.004, W = 0.07), cholesterol (*p* = 0.010, η^2^ = 0.05), and LDL (*p* < 0.019, η^2^ = 0.05). Post-intervention SBP and MAB values were found to be reduced in the LV-HIIT and 1-RT group. In the LV-HIIT group, DBP also decreased significantly from T-1 to T-2. Compared to the CON group, the reductions in SBP and MAB were significantly larger in the LV-HIIT (*p* = 0.002 and *p* < 0.001, respectively) and 1-RT group (*p* = 0.049 and *p* = 0.046, respectively). The LV-HIIT group experienced a greater reduction in MAB compared to the WB-EMS group (*p* = 0.002) and a greater reduction in DBP compared to the CON (*p* = 0.002) and the WB-EMS group (*p* = 0.001). Furthermore, post hoc tests revealed significant decreases in HbA_1c_ (LV-HIIT and WB-EMS), cholesterol (WB-EMS and CON), and LDL (1-RT and WB-EMS) from T-1 to T-2. Significant reductions in the MetS z-score were only observed in the LV-HIIT and 1-RT group. Improvements in the MetS z-score were significantly larger in the LV-HIIT group compared to the CON group (*p* = 0.004). All significant pre-/post-intervention changes for each group are shown in Table 3.

### 3.6. Cardiorespiratory Fitness Variables

Significant main effects of time and group-by-time interactions were found for relative VO_2max_ (*p* < 0.001, η^2^ = 0.17 and *p* < 0.001, η^2^ = 0.24, respectively), W_max_ (*p* < 0.001, η^2^ = 0.24 and *p* < 0.001, η^2^ = 0.40, respectively), and W_VT_ (*p* < 0.001, η^2^ = 0.16 and *p* < 0.001, η^2^ = 0.32, respectively). A significant group-by-time interaction was found for absolute VO_2max_ (*p* < 0.001, η^2^ = 0.24). Relative and absolute VO_2max_ only improved in the LV-HIIT group. W_max_ and W_VT_ increased in the LV-HIIT and WB-EMS groups. In contrast, significant reductions in absolute VO_2max_ and W_max_ were observed in the CON group from T-1 to T-2. Compared to the other groups, the LV-HIIT group experienced significantly greater improvements in relative VO_2max_ (*p* < 0.001 vs. CON, *p* = 0.043 vs. 1-RT and *p* = 0.034 vs. WB-EMS), absolute VO_2max_ (*p* < 0.001 vs. CON, *p* = 0.005 vs. 1-RT and *p* = 0.003 vs. WB-EMS), W_max_ (*p* < 0.001 vs. all groups), and W_VT_ (*p* < 0.001 vs. CON, *p* = 0.018 vs. 1-RT and *p* = 0.049 vs. WB-EMS). All group-specific changes in CRF variables are presented in Table 4.

## 4. Discussion

The present study was the first RCT to explore the effects of different very-low-volume exercise modalities on TL in a clinical cohort. The key finding was that TL significantly increased in obese MetS patients following 12 weeks of LV-HIIT but not after 1-RT and WB-EMS. Likewise, inflammation status, as quantified by decreased levels of CRP, IL-6, and LBP, only improved in the LV-HIIT group and remained unchanged in the other study groups. Moreover, despite similar amounts of weight loss in all groups, the MetS z-score only improved in patients performing LV-HIIT and 1-RT, while patients that were allocated to the WB-EMS and CON group did not experience significant changes in overall cardiometabolic health status.

Over the last decade, a body of research has demonstrated a link between the shortening of TL and a wide range of pathologic conditions, including cardiovascular disease [71,72,73], type 2 diabetes [71,74], obesity [75], MetS [14,15], and several cancer entities [76,77,78]. Decreased TL has also been reported to be associated with a poorer prognosis of cardiovascular [79] and cancer diseases [80,81] and related to an elevated risk of disease-specific and all-cause mortality [82]. Moreover, TL has been considered a “biological clock” to determine cellular and organismal ageing [16]. Thus, the exploration of potential strategies to preserve TL, including lifestyle modifications [83,84], has become an important and fruitful area of investigation in recent years.

Regarding lifestyle factors, there is growing evidence to support the beneficial impact of a physically active lifestyle on telomere maintenance. A number of large-scale observational studies, including data from the National Health and Nutrition Examination Survey (NHANES) [85,86,87], the Nurses Health Study (NHS) [88], the Berlin Aging Study II (BASE-II) [89], the Women’s Health Initiative (WHI) [90], and the Oulu Cohort 1945 [91], have documented that regular physical activity was associated with longer TL in a broad set of populations. Remarkably, the positive influence of physical activity has also been observed in twin studies that controlled for confounding variables, indicating that individuals who were less physically active exhibited shorter TL compared to their more active mono- or dizygotic siblings [92,93].

However, it is important to note that only a few RCTs have been conducted to evaluate the effects of targeted exercise interventions on TL. These trials have yielded inconsistent findings, with five studies reporting beneficial effects on TL [20,94,95,96,97] following a specific exercise intervention, while six studies did not observe significant changes in TL [98,99,100,101,102,103]. The conflicting results could mainly be due to the different exercise modalities used in previous RCTs, as seven studies applied continuous aerobic training regimens [20,94,97,98,100,101,102], three studies utilized interval training protocols [20,95,96], and three studies employed resistance training programs [20,99,103]. Accordingly, a recent systematic review on this topic [18] has highlighted that, at present, there is still a scarcity of knowledge on what type and intensity of exercise is most beneficial to achieve positive effects on TL. To date, only one RCT has been conducted to compare the influence of different exercise types on TL [20].

Our finding that only LV-HIIT (a type of cardiovascular exercise), but not the strength-based exercise types (1-RT and WB-EMS) improved TL is in line with the study by Werner et al. [20], who reported that TL increased after 6 months of aerobic endurance and interval training in previously sedentary but otherwise healthy individuals, but not with resistance training. Additionally, two other RCTs that implemented resistance training as a form of exercise intervention in breast cancer survivors [99] and in elderly individuals [103] did not observe a significant impact on TL. Thus, although it is undisputed that resistance training is an essential part of a well-rounded exercise routine as it is the most effective method to maintain/increase muscle mass and strength [104] and is independently associated with reduced mortality risk [105], our data support previous findings suggesting that resistance training alone may not be a sufficient stimulus to produce significant changes in TL. The reasons for these observations are not yet understood and remain to be investigated. It has been speculated that cardiovascular-based exercise modalities evoke higher heart rate responses than resistance training, leading to a higher vascular shear stress and, subsequently, a larger release of nitric oxide (NO) from the vessel walls [18,20], which has been shown to activate telomerase activity [106].

Furthermore, previous data from our laboratory indicate that LV-HIIT has greater efficacy for improving inflammatory indices in obese MetS patients compared to 1-RT and WB-EMS [33]. In agreement, in the present study, we observed significant reductions in CRP, IL-6, and LBP in the LV-HIIT group, while no changes were found in the other groups. Notably, CRP [107,108], IL-6 [109], and LBP [110] have been found to be significantly associated with various OS markers, such as 8-epi-prostaglandin-F_2α_, malondialdehyde (MDA), conjugated diene (CD), and endothelial nitric oxide synthase (eNOS). Thus, given the reported association between chronic inflammation [16,111], increased OS [13], and accelerated telomere shortening, the differential effects of the three low-volume exercise modalities on TL observed in the present study might also be linked to a greater anti-inflammatory and antioxidative potential of LV-HIIT when compared to 1-RT and WB-EMS.

Apart from the exercise type, the duration, intensity, and frequency of exercise are major components of a training protocol. Among these, intensity is regarded as the most critical determinant of the physiological responses to an exercise program [112]. Since previous studies have utilized various intensities in their exercise interventions, it is still unclear which training intensity provides the most beneficial impact on telomere dynamics [18]. Some recent investigations demonstrated, however, that individuals engaging in high-intensity endurance sports had longer TL when compared to those performing low- or moderate-intensity sports [113,114]. In this regard, it was noticeable that all previous RCTs applying high-intensity exercise protocols reported positive effects on TL [20,95,96], while studies using moderate-intensity aerobic training programs presented inconsistent findings [20,94,97,99,100,101,102], suggesting that cardiovascular exercise at higher intensities could potentially have more favourable effects on TL. The fact that the degree of CRF (typically quantified by VO_2max_) has been observed to be related to longer TL [91,115] supports this assumption, as higher exercise intensities are typically more effective for increasing VO_2max_ than lower intensities of exercise [116]. In this regard, high-intensity interval training, in particular, has been shown to be superior in improving VO_2max_ in healthy [117] and clinical cohorts [118]. Herein, it is important to note that an acute increase in OS can occur following physical exercise, particularly when performed at higher intensities [119]. At the same time, however, it has been postulated that exercise-induced increases in the production of ROS play an important, if not indispensable, role in physiological adaptations to training programs [119]. Accordingly, a recent review has concluded that regular participation in high-intensity exercise enhances antioxidative defence mechanisms in previously untrained individuals [120]. More specifically, it has been demonstrated that three weekly sessions of HIIT performed for 3 weeks improved antioxidant status indices, including catalase activity (CAT) and glutathione peroxidase (GPX) activity in healthy males [121]. Another study found that HIIT, performed over a period of 12 weeks, was more effective in decreasing MDA and increasing GPX compared to moderate-intensity continuous training in type 2 diabetes mellitus patients [122].

Given that time constraints are among the most frequently reported barriers to exercise participation [25,26,27], it is a crucial and novel result of this study that as little as 28 min of LV-HIIT per week—corresponding to only a fifth of the general physical activity recommendations [24]—appears to be effective enough to induce improvements in TL in obese MetS patients. Additionally, it should also be highlighted that the total weekly time effort for our LV-HIIT protocol was substantially lower (~40–80%) when compared to previous studies that have investigated the effects of interval training interventions on TL [20,95,96]. Consequently, to our knowledge, the total exercise volume per week used in the present study was the lowest to date that has shown a positive effect on TL. From a practical point of view, this finding is important, as it has been suggested that the generally recommended goal of achieving at least 150 min of physical activity per week [24] might be perceived as unrealistic and therefore demotivating for many individuals [123], especially for those with chronic diseases and/or physical limitations. Moreover, given that the risk of dropout from exercise programs has been found to be associated with the time effort required for an exercise program in previously sedentary populations [124], the present LV-HIIT protocol can be regarded as a feasible option for individuals who are not willing or able to engage in more exercise or used as an initial preparatory training step prior to higher-volume exercise programs.

When estimating the relevance of the observed TL change, we assume that the mean increases in the T/S ratio by ~0.05 units can be deemed clinically meaningful, as it has been previously reported that every 0.005 unit decline in T/S ratio is associated with a 1-year increment in biological age [125]. Apart from beneficial effects on TL, LV-HIIT also evoked favourable changes in VO_2max_ (2.9 mL/kg/min) and several cardiometabolic risk indices, including blood pressure (−10 mmHg SBP and −6 mmHg DBP, respectively), WC (−6 cm), and MetS z-score (−1.8 units), which can all be considered to have a clinically relevant impact [126,127,128]. These findings are in accordance with previous studies [33,34,35,36,37,38,39,40,41,42,43,44], indicating a robust effect of LV-HIIT on cardiometabolic health status. In this context, it is also noteworthy to emphasize that the changes in TL, inflammation, and cardiometabolic risk outcomes were irrespective of sex, age, and educational level. Only for the changes in WC a significant influence of sex was detected, with men showing significantly larger decreases than women. This finding is in line with previous findings and has been speculated to be due to a higher catecholamine-induced rate of free fatty acids mobilization from the visceral adipose tissue to the venous system in men compared to women [129,130].

Notably, the 1-RT group also exhibited a meaningful improvement in the MetS z-score (−1.1 units). This result is in line with previous research [41,131,132] and indicates that 1-RT can be deemed a viable low-volume exercise option for improving cardiometabolic health in obese patients with MetS. In contrast, WB-EMS improved some cardiometabolic indices, including WC and cholesterol levels, but it was not sufficiently effective to improve overall MetS severity status. This observation is relevant because the MetS z-score has been suggested to be more accurate in predicting the patient’s risk of developing CVD in the future compared to merely adding up single cardiometabolic risk factors [54]. In recent years, WB-EMS has been widely advertised as an effective and time-saving strength exercise alternative to conventional resistance training. Indeed, there is a solid body of evidence indicating that WB-EMS can be an effective exercise option for improving muscle strength and body composition in trained individuals and untrained but otherwise healthy populations [32,66]. However, the effects of WB-EMS on cardiometabolic health outcomes are less well studied and still inconclusive [67]. Our present results are in agreement with previous studies [33,41] and suggest that traditional resistance training is more powerful in improving cardiometabolic health status—at least in patients with pre-existing cardiometabolic disorders.

### Strengths and Limitations

This study has several notable strengths. First, the present investigation was the first that has evaluated the effects of different very-low-volume exercise modalities on TL in a clinical cohort. Given the growing popularity of time-saving training protocols and their increasing application in clinical settings, our results provide important novel findings with practical implications for those involved in designing and implementing exercise programs for obese patients. The presented low-volume exercise protocols are simple, easy to replicate, and feasible for individuals at increased cardiometabolic risk, as indicated by high attendance rates, low dropout rates, and the absence of exercise-related adverse events. Second, we used well-established and accepted diagnostic measures to quantify our outcomes, including, for example, the qPCR method for TL determination or CPET (the gold standard assessment of VO_2max_), which were all conducted by blinded assessors. All examinations were based on current guidelines and were carried out under strictly standardized conditions. Third, the monitoring of patients’ food intake within the framework of standard-care nutritional counselling showed that the nutritional intakes were similar between groups, indicating that our results were not biased by dietary patterns.

However, there are also some potential limitations that should be considered when interpreting our results. First, we note that we did not measure specific markers of OS or antioxidative capacity in this study. Thus, our interpretations regarding the potential relationship between the observed increase in TL in the LV-HIIT group and improved OS remain speculative. However, the significant decrease in CRP, IL-6, and LBP following LV-HIIT, which are strongly associated with markers of OS-markers, supports a probable link between decreased inflammation, OS load, and improved TL. Second, we point out that a higher number of our study participants were women (~67%), which could limit the generalizability of the results. A higher proportion of female participants in weight loss interventions appears to be a common phenomenon that has also been observed in other studies [133,134,135]. However, given that the sex distribution was not significantly different between groups and due to the lack of a sex effect on changes in primary and secondary outcomes (except for waist circumference), we do not expect a meaningful impact of this limitation on our major research question (i.e., the comparison of the different low-volume exercise protocols in their effects on TL). Third, we acknowledge that the training protocols were performed in a well-controlled clinical setting and that all exercise sessions were carefully supervised. Although there is growing evidence to support the safe application of exercise interventions, including high-intensity interval training [136], for example, in clinical populations, the feasibility and efficacy of our low-volume exercise protocols will need to be critically evaluated in other conditions outside of the research setting (e.g., in rehabilitation and health centres) to support the generalizability of study findings. Based on our findings, including high attendance rates and an excellent adverse event profile, we expect that the low-volume exercise programs used in this study will also exhibit high tolerability and acceptance in “real-world” conditions—provided that an appropriate medical clearance has been carried out beforehand. Lastly, given that the present study lasted 12 weeks, the longer-term effects of very-low-volume exercise protocols on TL remain to be investigated in the future.

## 5. Conclusions

This is the first RCT to investigate the impact of different very-low-volume exercise modalities on TL in a clinical cohort. We provide novel evidence that LV-HIIT, requiring less than 30 min of effort per week, shows the potential to increase TL in obese MetS patients, potentially due to improvements in OS-related inflammatory markers. Additionally, LV-HIIT induced clinically meaningful improvements in CRF and several cardiometabolic risk outcomes. One-set RT and WB-EMS improved some cardiometabolic indices (with 1-RT showing greater efficiency vs. WB-EMS in improving MetS severity) but did not significantly affect TL, indicating that very-low-volume exercise modalities have differential effects on telomere dynamics and cardiometabolic health. Therefore, low-volume exercise programs targeting the prevention of cellular ageing should primarily focus on cardiovascular-based training modalities. Given that low-volume resistance training types do not seem to have a meaningful impact on TL, they should rather be implemented as a complement to a well-rounded exercise routine to address muscle strength but not as a substitute for cardiovascular training.

## Figures and Tables

**Table 1:** Patients’ baseline characteristics.

Variable	LV-HIIT (*n* = 52)	1-RT (*n* = 19)	WB-EMS (*n* = 22)	CON (*n* = 30)
Age (years)	53 (49 to 57)	55 (50 to 60)	52 (47 to 57)	51 (46 to 57)
Sex, male/female (*n*)	21/31	4/15	8/14	8/22
Educational level				
Low ^1^ (*n*)	13 (25%)	4 (21%)	7 (32%)	9 (30%)
Medium ^2^ (*n*)	19 (37%)	9 (47%)	8 (36%)	12 (40%)
High ^3^ (*n*)	20 (38%)	6 (32%)	7 (32%)	9 (30%)
Anthropometric variables				
Body weight (kg)	108.6 (102.3 to 114.9)	106.0 (92.4 to 119.5)	108.5 (98.9 to 116.3)	105.9 (98.7 to 112.9)
Body mass index (kg/m^2^)	37.5 (35.6 to 39.4)	38.1 (34.3 to 42.0)	37.7 (35.6 to 39.6)	37.4 (35.4 to 39.4)
Body fat (%)	44.0 (42.0 to 45.9)	46.6 (44.1 to 49.1)	44.0 (42.0 to 46.4)	45.2 (42.9 to 48.0)
Waist circumference (cm)	112 (108 to 116)	111 (103 to 119)	114 (109 to 119)	110 (106 to 114)
Blood pressure				
SBP (mmHg)	140 (135 to 144)	146 (138 to 154)	137 (130 to 143)	135 (136 to 142)
DBP (mmHg)	89 (87 to 92)	90 (83 to 97)	87 (83 to 91)	86 (82 to 90)
MAB (mmHg)	106 (103 to 109)	109 (103 to 115)	103 (99 to 108)	103 (98 to 107)
Inflammatory markers				
CRP (mg/L)	5.5 (4.1 to 7.0)	5.1 (2.2 to 8.0)	3.9 (2.8 to 5.1)	3.9 (2.6 to 5.2)
IL-1β (pg/mL)	4.8 (3.5 to 6.1)	8.3 (3.4 to 13.1)	8.9 (7.1 to 10.7)	6.7 (4.8 to 8.6)
IL-6 (pg/mL)	3.0 (2.3 to 3.7)	2.8 (1.9 to 3.7)	2.6 (2.0 to 3.2)	3.0 (2.3 to 3.8)
INFγ (pg/mL)	8.6 ± 5.5	7.0 ± 4.4	7.3 ± 2.9	8.1 ± 4.5
Adiponectin (µg/mL)	2.5 (2.0 to 3.0)	4.1 (2.4 to 5.8)	2.5 (1.8 to 3.1)	2.7 (2.1 to 3.3)
LBP (ng/mL)	12.5 ± 3.4	30.3 ± 7.8	23.0 ± 6.4	16.2 ± 8.4
Clinical chemistry				
Glucose (mg/dL)	104 (98 to 110)	98 (91 to 106)	104 (98 to 109)	100 (93 to 107)
HbA_1c_ (%)	5.7 (5.5 to 5.9)	5.6 (5.4 to 5.8)	5.7 (5.4 to 5.9)	5.6 (5.3 to 5.9)
Triglycerides (mg/dL)	136 (117 to 156)	123 (105 to 141)	132 (105 to 159)	155 (125 to 183)
Cholesterol (mg/dL)	213 (203 to 223)	229 (216 to 243)	222 (207 to 238)	228 (213 to 246)
HDL (mg/dL)	49 (46 to 52)	59 (51 to 67)	55 (49 to 61)	55 (50 to 60)
LDL (mg/dL)	142 (134 to 150)	150 (139 to 161)	146 (133 to 158)	147 (135 to 160)
MetS z-score	2.6 (1.6 to 3.7)	2.1 (1.3 to 3.9)	2.3 (1.1 to 3.3)	1.9 (1.2 to 3.0)
CPET variables				
VO_2max_ (L)	2.31 (2.13 to 2.49)	2.10 (1.83 to2.37)	2.30 (2.01 to 2.55)	2.18 (1.86 to 2.49)
VO_2max_ (mL/kg/min)	21.5 (20.1 to 23.1)	20.3 (17.9 to 22.7)	21.1 (19.2 to 24.1)	20.4 (17.9 to 23.0)
W_max_ (W)	146 (134 to 157)	158 (139 to 177)	167 (142 to 189)	153 (130 to 176)
W_VT_ (W)	58 (51 to 65)	78 (65 to 90)	72 (63 to 82)	67 (56 to 80)
Dietary inakes				
Energy (kcal)	2144 (1885 to 2415)	2297 (1898 to 2696)	2448 (2127 to 2770)	2249 (1936 to 2564)
Protein (g/kg)	0.9 (0.7 to 1.1)	1.0 (0.8 to 1.1)	1.0 (0.8 to 1.1)	0.9 (0.7 to 1.1)
Fat (g/kg)	0.8 (0.7 to 0.9)	0.9 (0.8 to 1.0)	1.0 (0.8 to 1.2)	0.9 (0.8 to 1.1)
Carbohydrates (g/kg)	2.0 (1.8 to 2.3)	2.1 (1.9 to 2.4)	2.4 (1.9 to 2.8)	2.1 (1.7 to 2.5)

Data are shown as mean and 95% CIs. ^1^ Lower or intermedium secondary school, ^2^ high school, ^3^ college or university. SBP/DBP = systolic/diastolic blood pressure, MAB = mean arterial blood pressure, CRP = C-reactive protein, IL-1β = interleukine-1 beta, IL-6 = interleukine-6, IFNγ = interfereonegamma, LBP = lipopolysaccharide-binding protein, HbA_1c_ = glycosylated haemoglobin A_1c_, HDL/LDL = high-/low-density lipoprotein cholesterol, MetS z-score = metabolic syndrome severity score, CPET = cardiopulmonary exercise testing, VO_2max_ = maximal oxygen uptake, W_max_ = maximal power output, W_VT_ = power output at the ventilatory threshold.

**Table 2:** Changes (Δ) in patients’ inflammatory markers between T-1 and T-2.

Variable	LV-HIIT (*n* = 52)	1-RT (*n* = 19)	WB-EMS (*n* = 22)	CON (*n* = 30)
CRP (mg/L)	−1.5 (−2.8 to −0.2) *	0 (−0.6 to 0.6)	−0.4 (−1.1 to 0.4)	1.5 (−1.1 to 4.2)
*p*-value	0.015	0.943	0.311	0.243
IL-6 (pg/mL)	−0.6 (−1.1 to −0.1) *	−0.2 (−0.8 to 0.3)	−0.3 (−0.8 to 0.2)	0.7 (−0.4 to 1.8)
*p*-value	0.014	0.410	0.242	0.182
LBP (ng/mL)	−1.4 (−2.5 to −0.3)	−1.2 (−3.0 to 0.7)	−0.2 (−1.2 to 0.7)	0.2 (−0.9 to 1.3)
*p*-value	0.009	0.193	0.604	0.696

Data are shown as mean and 95% CIs. CRP = C-reactive protein, IL-6 = interleukine-6, LBP = lipopolysaccharide-binding protein. In case of significant main effects in the 2-way repeated measures ANOVA, comparisons between groups were tested using 1-way ANOVAs followed by Holm–Sidak post hoc tests. Within-group changes (Δ) between T-1 and T-2 were evaluated using paired *t*-tests. *p*-values below Δ-values = significant within-group changes; * (*p* < 0.05) = significant difference vs. CON.

**Table 3:** Changes (Δ) in patients’ anthropometric and cardiometabolic data between T-1 and T-2.

Variable	LV-HIIT (*n* = 52)	1-RT (*n* = 19)	WB-EMS (*n* = 22)	CON (*n* = 30)
Anthropometric variables				
Body weight (kg)	−3.8 (−5.1 to −2.5)	−3.0 (−5.6 to −0.5)	−4.2 (−5.8 to −2.5)	−2.8 (−3.7 to −1.9)
*p*-value	<0.001	0.012	<0.001	<0.001
Body mass index (kg/m^2^)	−1.3 (−1.7 to −0.9)	−1.1 (−2.0 to −0.2)	−1.4 (−2.0 to −0.9)	−1.0 (−1.3 to −0.7)
*p*-value	<0.001	0.012	<0.001	<0.001
Body fat (%)	−1.4 (−2.1 to −0.6)	−1.2 (−2.0 to −0.4)	−0.9 (−1.6 to −0.3)	−1.0 (−1.8 to −0.3)
*p*-value	<0.001	0.002	0.005	0.003
Waist circumference (cm)	−6 (−7 to −4) **	−5 (−8 to −2)	−3 (−5 to −1)	−1 (−3 to 1)
*p*-value	<0.001	0.001	0.001	0.054
Blood pressure				
SBP (mmHg)	−10 (−14 to −7) **	−9 (−17 to −1)	−3 (−10 to 4)	1 (−4 to 6)
*p*-value	<0.001	0.024	0.193	0.335
DBP (mmHg)	−6 (−8 to −4) ** ^##^	−4 (−11 to −2)	2 (−1 to 5)	0 (−3 to 3)
*p*-value	<0.001	0.085	0.056	0.769
MAB (mmHg)	−8 (−10 to −5) *** ^##^	−6 (−12 to −1) *	0 (−2 to 2)	1 (−3 to 4)
*p*-value	<0.001	0.018	0.468	0.694
Clinical chemistry				
HbA_1c_ (%)	−0.1 (−0.2 to 0)	0 (−0.1 to 0)	−0.1 (−0.2 to 0)	0 (−0.1 to 0)
*p*-value	0.036	0.240	0.010	0.113
Cholesterol (mg/dL)	−1 (−8 to 7)	−7 (−16 to 2)	−8 (−16 to −1)	−9 (−18 to 0)
*p*-value	0.438	0.054	0.017	0.023
LDL (mg/dL)	−1 (−7 to 6)	−7 (−15 to 1)	−6 (−14 to 1)	−4 (−9 to 3)
*p*-value	0.453	0.033	0.043	0.114
MetS z-score	−1.8 (−2.3 to −1.4) **	−1.1 (−2.1 to −0.1)	−0.4 (−0.9 to 0.2)	−0.5 (−1.2 to 0.2)
*p*-value	<0.001	0.014	0.080	0.087

Data are shown as means and 95% CIs. T-1 = pre-intervention, T-2 = post-intervention, SBP/DBP = systolic/diastolic blood pressure, MAB = mean arterial blood pressure, HbA_1c_ = glycosylated hemoglobin A_1c_, HDL/LDL = high-/low-density lipoprotein cholesterol, MetS z-score = metabolic syndrome severity score. In case of significant main effects in the 2-way repeated measures ANOVA or the Friedman 2-way analysis of variance by ranks (HbA_1c_), comparisons between groups were tested using 1-way ANOVAs followed by Holm–Sidak post hoc tests or Kruskal–Wallis tests followed by Dunn’s Bonferroni post hoc tests (HbA_1c_), respectively. Within-group changes (Δ) between T-1 and T-2 were evaluated using paired *t*-tests or Wilcoxon tests (HbA_1c_), respectively. *p*-values below Δ-values = significant within-group changes; * (*p* < 0.05), ** (*p* < 0.001), *** (*p* < 0.001) = significant difference vs. CON; ^##^ (*p* < 0.01) = significant difference vs. WB-EMS.

**Table 4:** Changes (Δ) in patients’ CPET data after intervention period.

Variable	LV-HIIT (*n* = 52)	1-RT (*n* = 19)	WB-EMS (*n* = 22)	CON (*n* = 30)
CPET variables				
VO_2max_ (L)	0.21 (0.14 to 0.27) *** ^††^	0.08 (−0.07 to 0.23)	0.05 (−0.04 to 0.13)	−0.13 (−2.5 to −0.01)
*p*-value	<0.001	0.148	0.266	0.014
VO_2max_ (mL/kg/min)	2.9 (2.1 to 3.7) *** ^†^	1.2 (−0.1 to 2.4)	1.4 (0.5 to 3.1)	−0.2 (−0.8 to 0.4)
*p*-value	<0.001	0.068	0.055	0.519
W_max_ (W)	22 (18 to 26) *** ^†††^	6 (−1 to 13) *	8 (3 to 12) *	−4 (−8 to −0)
*p*-value	<0.001	0.090	0.003	0.020
W_VT_ (W)	21 (16 to 25) *** ^#^	3 (−4 to 11)	7 (2 to 12)	−2 (−6 to 2)
*p*-value	<0.001	0.370	0.010	0.345

Data are shown as mean 95% CIs. CPET = cardiopulmonary exercise testing, VO_2max_ = maximal oxygen uptake, W_max_ = maximal power output, W_VT_ = power output at the ventilatory threshold. In case of significant main effects in the 2-way repeated measures ANOVA, comparisons between groups were tested using 1-way ANOVAs followed by Holm–Sidak post hoc tests. Within-group changes (Δ) between T-1 and T-2 were evaluated using paired *t*-tests. *p*-values below Δ-values = significant within-group changes; * (*p* < 0.05), *** (*p* < 0.001) = significant difference vs. CON; † (*p* < 0.05), †† (*p* < 0.01), ††† (*p* < 0.001) = significant difference vs. 1-RT and WB-EMS; ^#^ (*p* < 0.05) = significant difference vs. 1-RT.

## Data Availability

The datasets generated and analyzed during the current study are not publicly available but are available from the corresponding author upon reasonable request.
